# Efficacy of an impression disinfectant solution after repeated use: An *in vitro* study

**DOI:** 10.1016/j.heliyon.2023.e23792

**Published:** 2023-12-15

**Authors:** Simon Chukwu, Alan Munn, Jennifer C. Wilson, Hadeel Ibrahim, Dean Gosling, Robert M. Love, Mahmoud M. Bakr

**Affiliations:** a Clinical Dental technologist, L & T Dental Laboratory Bridgend, Wales, UK; b Senior Lecturer in Biochemistry, School of Pharmacy and Medical Sciences, Griffith University, Queensland, Australia; c Associate Professor, School of Pharmacy and Medical Sciences, Griffith University, Queensland, Australia; d Lecturer in Prosthodontics, University of Melbourne, Prosthodontist at the Royal Dental Hospital of Melbourne, Australia; e Advanced Scientist - Laboratory Operations Manager, Queensland Public Health and Scientific Services (QPHaSS), Queensland Health, Queensland, Australia; f Dean of Dentistry, School of Medicine and Dentistry, Griffith University, Queensland, Australia; g Director of Clinical Education, Senior lecturer in General Dental Practice, School of Medicine and Dentistry, Griffith University, Queensland, Australia

## Abstract

**Statement of problem:**

There are very few studies using Benzalkonium Chloride (BAC) as an active disinfection agent for immersion techniques and there are no studies investigating the efficacy of repeated use of a disinfectant solution.

**Purpose:**

This study evaluated an impression disinfectant by testing bacterial contamination of disinfectant batches used in a clinical setting after repeated use.

**Materials and methods:**

Liquid samples were collected from impression disinfectant solutions used to disinfect dental impressions taken at a university dental clinic. The experimental samples (500 ml from 1 L of solution) were collected from teaching and professional clinics and the in-house commercial processing laboratory and stored at room temperature each day of clinic operation over five weeks. To determine to what extent the disinfectant efficacy of the active product decreased over time, the following tests were carried out: a. Inoculation b. Gram staining technique c. Matrix Assisted Laser Desorption/Ionization Mass spectrometry (MALDI- MS). Microbial growth was monitored and photographed. A culture revival was made from colonies grown on sheep blood agar, to isolate pure colonies incubated for 24 h at 37 °C. Each morphologically distinct type of colony was gram stained and MALDI spectrometry analysis was performed using the VITEK MS (BioMerieux Inc.)

**Results:**

Evidence of growth of bacteria was detected in teaching clinics’ samples, and no growth from the professional clinic or the commercial laboratory.

**Conclusions:**

The study demonstrated that the impression disinfectant solution tested is effective against common oral bacteria, despite some rare species such as *Bacillus circulans, Bacillus horneckiae, Bacillus altitudinis/pumilus* and *Bacillus cereus* showing evidence of survival in solutions used for disinfection of impressions. However, in a high use teaching clinic environment its efficacy deteriorated. Though a second level disinfection protocol in the commercial laboratory-maintained impression disinfection.

## 1 Clinical implications

The present study demonstrates the importance of having a second level disinfection protocol in dental laboratories to ensure the highest levels of impression disinfection and the absence of spores and/or uncommon bacterial species. Furthermore, following the manufacturer's instructions in terms of pre-rinsing impressions for 3 min before disinfection is an important factor in maintaining the efficacy of the disinfecting solution. Finally, dental personnel's level of experience as well as the daily impression number/load affected the efficacy of the disinfecting solution. The present study informs clinicians that the Cavex Impresafe disinfecting solution needs to be changed more frequently (daily rather than weekly) in larger dental facilities with higher impression turnover per day.

## 2 Introduction

Antimicrobial disinfection has been a critical step in clinical dentistry and dental research aiming to ensure the health and safety of patients and practitioners as well as improving the quality of treatment outcomes [1,2]. Prevention of transmission of pathogens from patients to the dental team is at the cornerstone of practicing dentistry. Studies have shown that dental impressions can harbor various pathogenic microorganisms [[3], [4], [5]]. Benzalkonium chloride (BAC) is a common solution used in the dental office to disinfect dental impressions and prevent the transfer of microorganisms from patients to dental and laboratory personnel. One advantage of detergent-like active agents -such as BAC- over oxidising active agents - such as sodium hypochlorite-is that the latter lose disinfectant power over time (even if not used) whereas the former do not degrade over time [6]. However, despite the low degradability of BAC, disinfectant solutions using BAC still lose power with repeated use owing to three main reasons. First, the more times a solution is used the more dead bacteria are left in the solution [7]. Accumulation of dead bacteria creates turbidity and reduces disinfectant power. Second, the active molecules of BAC have a negative charge, some impression materials neutralise this charge reducing the disinfectant power faster. Finally, the surfaces of some impression materials absorb active molecules of BAC whenever an impression is submerged for effective disinfection to take place. Hence, the more the solution is used, the fewer active molecules are left, and the less disinfectant power remains [8].

Despite the widespread use of immersion disinfection procedures there is paucity of literature using BAC as active agent in immersion techniques [9]. A common theme in published studies was to determine the thoroughness of disinfection of dental impressions [10,11]. The manufacturer of one of the commonly used BAC solutions, Cavex Impresafe, claims that Cavex Impresafe can be used for 7 days or until visibly contaminated. There is a lack of studies investigating this claim after repeated use of this BAC disinfectant. Moreover, despite the negative impact that recurrent use of the solution has on its disinfectant power, there are no studies analysing changes in the disinfectant efficacy of BAC with repeated uses of the solution and different impression materials. Manufacturers of commercial products with BAC provide an indicative immersion time of 3 min per piece, encouraging repeated use of the same solution. However, it is unclear when the solution should be changed. The advice provided by the manufacturer is ambiguous, it refers to generic factors such as how "dirty" the solution is and is not supported by empirical evidence. Within the literature, there is a gap in the existing research with only a few studies using BAC as an active agent in dentistry and very few investigations on the repeated use of the solution. The current study fills a gap in the literature as it tests the efficacy of a BAC disinfectant (Cavex Impresafe) in different clinical and laboratory settings, removable prosthetics teaching clinic, general dentistry teaching clinic, professional dental clinic and a commercial dental laboratory over different time points of usage. The aim of this study is to demonstrate whether bacteria can colonise or grow in a commercial disinfectant solution, Cavex ImpreSafe, as used during disinfection of dental impression materials at different time intervals.

## 3 Materials and methods

### 3.1 Sample collection and testing method

Liquid samples were collected from Cavex ImpreSafe (Cavex Holland BV, Haarlem, The Netherlands) disinfectant solution consisting of 3 % quaternary ammonium compound (benzalkonium chloride) that had been used to disinfect primary and secondary impressions collected from patients at the Griffith University Dental Clinic Gold Coast Campus. The samples excluded dentures that had been returned for repairs or relining. Cavex Impresafe contains the following ingredients, Quaternair ammonium salt (30%w), surfactant (<1%w), cleaning booster (<1%w), pigment blue in the form of spores and water (70%w). The following impressions were used in the Griffith University Dental Clinic Gold Coast Campus.

• **Alginate impression material** – Blueprint® Xcreme (Dentsply Sirona, Konstanz, Germany)
• **Polysiloxane condensation silicone impression material** – Optosil® Confort® Putty (Kulzer GmbH, Hanau, Germany)
• **Vinyl polysiloxane impression material**

Extrude (Kerr™, Romulus MI, USA)
Correct PlusTM. (Pentron Clinical, Orange, CA, USA)
Take 1® Advanced (KerrTM., Orange, CA, USA)
Flexitime® (Kulzer GmbH, Hanau, Germany)

• **Polyether impression material** – Impregum™ Penta™ (3 M Deutschland GmbH, Neuss, Germany)
• **Zinc oxide and Eugenol impression material** – White (SS White Group, Gloucester, England)

Cavex Impresafe contains the following ingredients, Quaternair ammonium salt (30%w), surfactant (<1%w), cleaning booster (<1%w), pigment blue in the form of spores and water (70%w). The disinfectant solution used in this study was prepared from Cavex Impresafe according to the manufacturer's instructions; each 30 ml (about 1.01 oz) of the concentrate was diluted to 1L with water. The disinfection protocol comprised rinsing the impressions under tap water for 15 s. The impressions were then immersed in the disinfectant for 3 min following the manufacturer's recommendations. After 3 min, the impressions were removed from the disinfectant solution and rinsed off with water for 15 s before bagging.

To determine the efficacy of used Cavex ImpreSafe disinfectant solution in subsequent intervals the following oral bacteria (Streptococci, Lactobacilli, Staphylococci, Corynebacterium, *Actinobacillius actinomycetemcomitans* and different anaerobic especially Bacteroides) were tested for by culturing the collected samples from the teaching clinics, professional dental clinic and the commercial dental laboratory on various culture media for five weeks on a daily basis. This meant a total of 25 samples for each of the areas accounting for daily samples five days per week (Monday-Friday) for 5 weeks.The following tests were performed: Inoculation, gram staining technique and Matrix Assisted Laser Desorption/Ionization Time-of-Flight Mass Spectrometry (MALDI-TOF MS). The tests were completed in the Gold Coast University Hospital (GCUH) Laboratory – Pathology Queensland that is accredited to ISO 15189.

The MALDI-TOF MS technique is used for early identification of groups of bacteria that are difficult to culture [[12], [13], [14]]. The samples were prepared as per below for the MALDI-TOF MS technique.

• The samples were applied to the target slide using a Pick Me Pen and Nib, toothpick or disposable loop touch a colony or pure growth without picking up agar when sampling the colony.
• A small amount of the samples was deposited, and thin layers of the samples were smeared onto the center of the slides.
• Using a calibrated pipette, 1 μL of matrix was applied promptly on the target slide.
• The matrix/microorganism suspension was allowed to air dry completely. This is important to allow the matrix crystals to become visible as a yellowish film, and this is necessary for the measurement to proceed.

The samples obtained from the dental clinic were inoculated on petri dishes of sheep blood agar and tryptic soy agar and incubated for 14 days at 37 °C. The samples were inspected and checked daily for any microbial growth. Gram staining technique was used to differentiate two major classifications of bacteria: gram-positive and gram-negative bacteria [15]. The smear for Gram stain was prepared as per the steps below.

• Fixed smear on glass slides were prepared from the clinical samples (1 μl Loop) on solid media.
• The smears were air dried after preparation and fixed to the slides by placing on a heating block until fluid has dried completely, and then allowing the slide to cool.
• The slides were placed on a staining rack and the surface was overlayed with crystal violet solution for 1 min followed by washing with tap water, overlay with iodine for 1 min then washed again with tap water.
• Decolorizer (ethyl alcohol) was applied until no more violet color washes off, then washed immediately with tap water.
• The slides were overlayed with dilute Carbol-fuchsin/safranin counterstain for 1 min, then washed with tap water and left in an upright position to dry.

The design of this experiment was based on concepts from Bergey's Manual of Determinative Bacteriology [16] to closely resemble what happens in practice when impressions are disinfected.

### 3.2 Daily protocol for measurement of reduction of disinfecting power with repeated uses

A control batch of disinfectant was prepared for use as a comparison to the experimental samples. The experimental samples were collected from each clinic and the commercial laboratory, for each day of clinic operation over five weeks. For each operating day, the disinfectant would be used to clean impressions taken during three or more clinical/teaching sessions irrespective of the number of impressions in each session. It should be noted that impressions received by the commercial laboratory would have been already disinfected in the dental clinic. Therefore, samples collected from the commercial laboratory are used to disinfect impressions as a second level disinfection protocol.

### 3.3 Isolation and identification of microorganisms

Of the samples collected from the Griffith University Dental clinic, 50 ml aliquots were pipetted into centrifuge tubes and spun for 10 min at 4700 rpm at 20 °C. The pellet sediment was retained, and the supernatant discarded. The pellet was washed twice with 5 ml of autoclaved-distilled water and centrifuged at 4700 rpm for 5 min at 20 °C. This step diluted out the disinfectant agent, reducing the incidence of chemical haemolysis when plated on sheep blood agar. 100 μl samples were obtained with a micropipette, and inoculated onto tryptic soy agar, and sheep blood agar plates using disposal spreaders. The plates were incubated at 37 °C for 14 days and inspected daily. A culture revival was made from colonies grown on sheep blood agar, to isolate pure colonies. Samples of each colony were streaked out on to a clean plate of sheep blood agar and tryptic soy agar. The streak plates were incubated for 24 h at 37 °C. The primary isolation plate was refrigerated for future use. Microbial growth from the secondary plate was streaked out on selective and differential media. MacConkey agar No 2, 3 and Mannitol salt plates were incubated for 24 h at 37 °C. Each morphologically distinct type of colony was sampled. Gram stain slides were prepared and MALDI spectrometry analysis was performed using the Bruker Biotyper (Bruker Daltonics) system. Using MALDI-TOF analysis, the Bruker Biotyper identifies microorganisms by comparing mass spectrometry data against a database of microorganism profiles.

## 4 Results

### 4.1 The isolation of microorganisms from Cavex Impresafe disinfectant

**Fig. 1:**
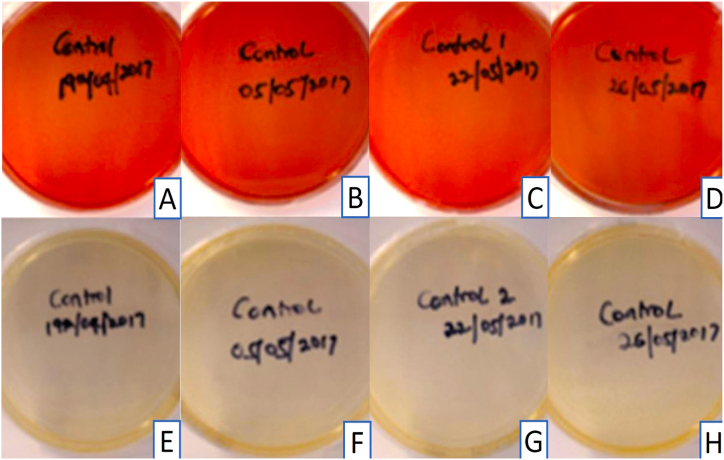
Microbial growth of the control group after 14 days of incubation at 37 °C. A-D, Showing no microbial growth on sheep blood agar plates. E-H, Showing no microbial growth on tryptic soy agar plates.

**Fig. 2:**
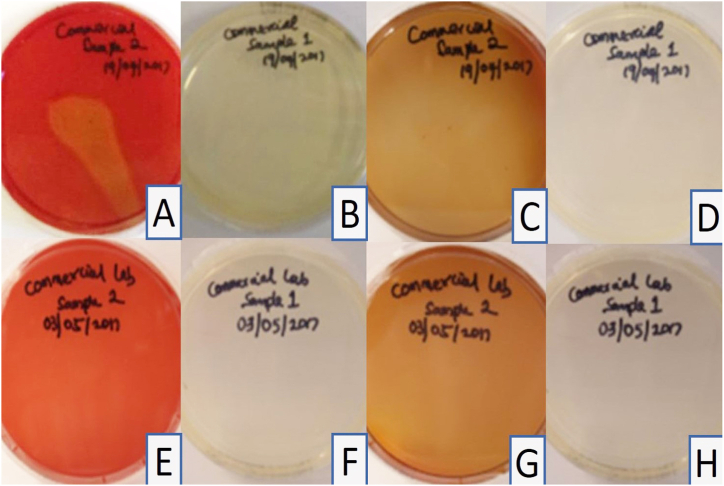
Commercial dental laboratory group samples. A, E, No microbial growth on sheep blood agar plates after 24 h of incubation at 37 °C. B, F Tryptic soy plates after 24 h of incubation at 37 °C. C, G Sheep blood agar plates after 14 days of incubation at 37 °C. D, H, tryptic soy agar plates after 14 days of incubation at 37 °C.

**Fig. 3:**
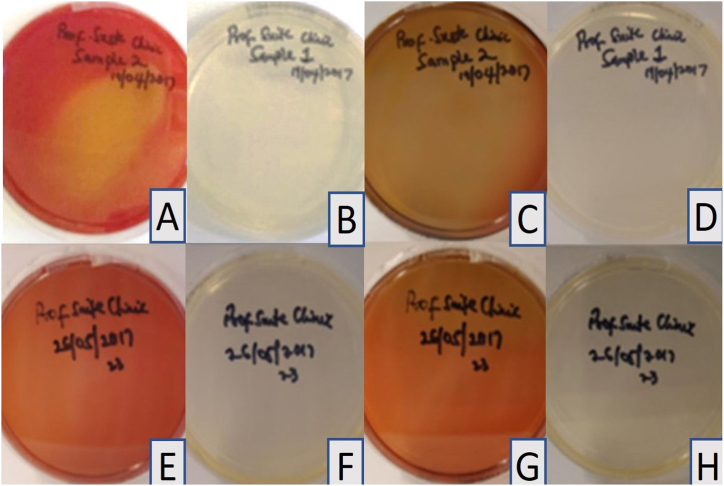
Professional dental clinic group samples showing no microbial growth. A, E, Sheep blood agar plates after 24 h of incubation at 37 °C. B, F Tryptic soy agar plates after 24 h of incubation at 37 °C. C, G Sheep blood agar plates after 14 days of incubation at 37 °C. D, H Tryptic soy agar plates after 14 days of incubation at 37 °C.

**Fig. 4:**
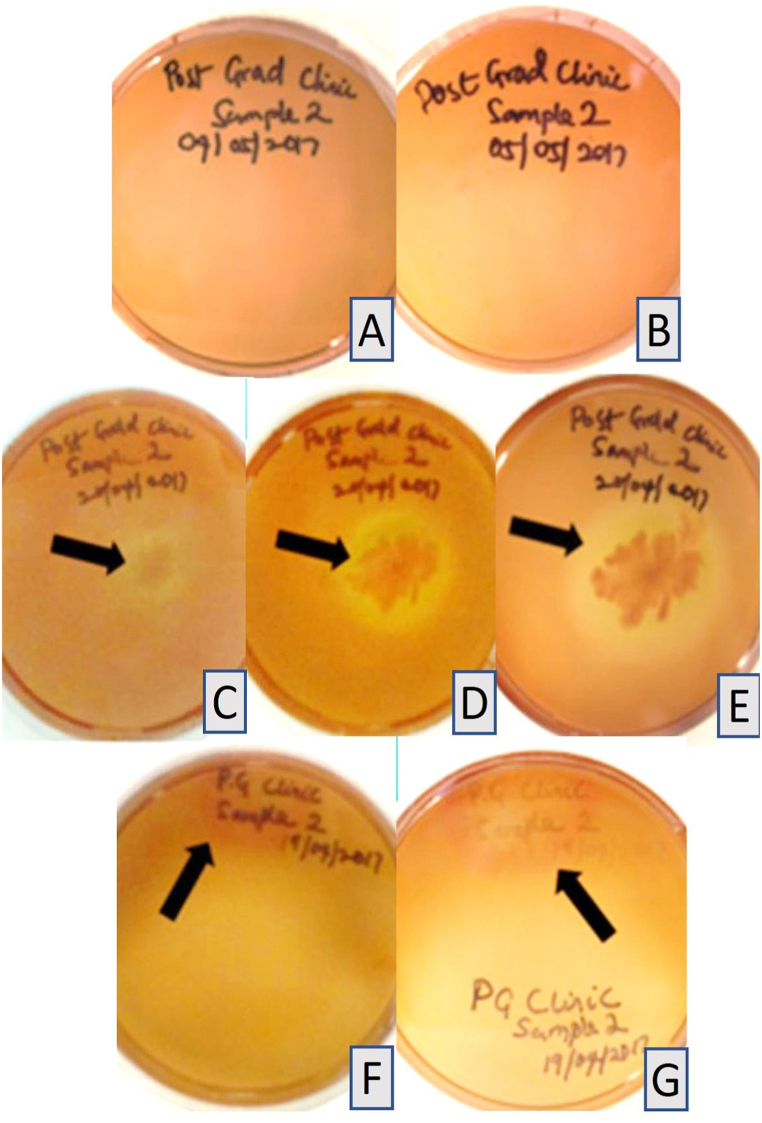
Removable prosthetics teaching clinic group samples. A, B, Showing no bacterial growth on sheep blood agar plates after 24 h of incubation at 37 °C. C, Removable prosthetics teaching clinic sample inoculated on sheep blood agar at 37 °C with microbial growth found after 3 days. D, Further growth of the same bacterial colony after 6 days. E, after 8 days. F, Showing a removable prosthetics teaching clinic sample inoculated on sheep blood agar at 37 °C with microbial growth found after 7 days. G, further growth of the same bacterial colony after 9 days. * The black arrows indicate the microbial growth.

**Fig. 5:**
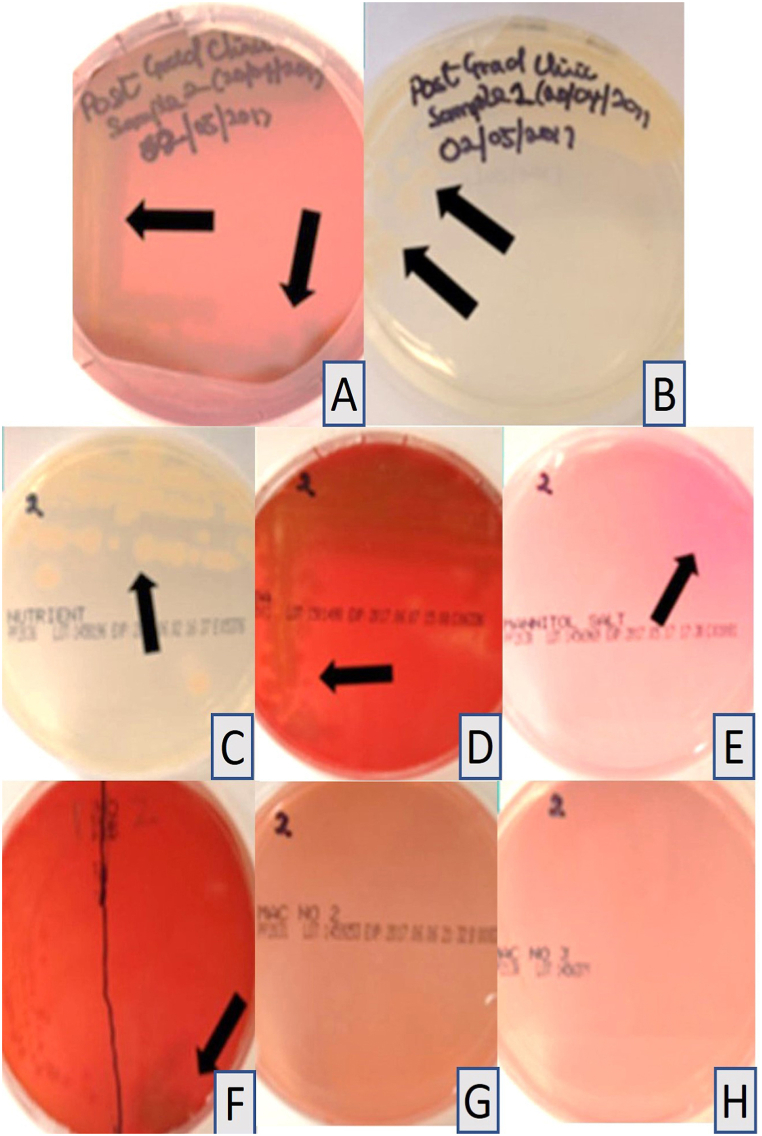
Selective and differential media of removable prosthetics teaching clinic sample after 24 h of incubation at 37 °C. A, B, Culture revival of the removable prosthetics teaching clinic sample streaked out on to a clean sheep blood agar and tryptic soy agar showing microbial growth after 24 h of incubation at 37 °C. C–H, C, Nutrient agar plate. D, Columbia horse blood agar plate. E, Mannitol salt agar plate. F, Columbia horse + Nalidixic acid and Colistin agar plate showing microbial growth. G, MacConkey No 2 - agar plate and H, MacConkey No 3 - agar plate showed no microbial growth. This indicates that the microorganism is not a lactose-fermenting organism. * The black arrows indicate microbial growth.

**Fig. 6:**
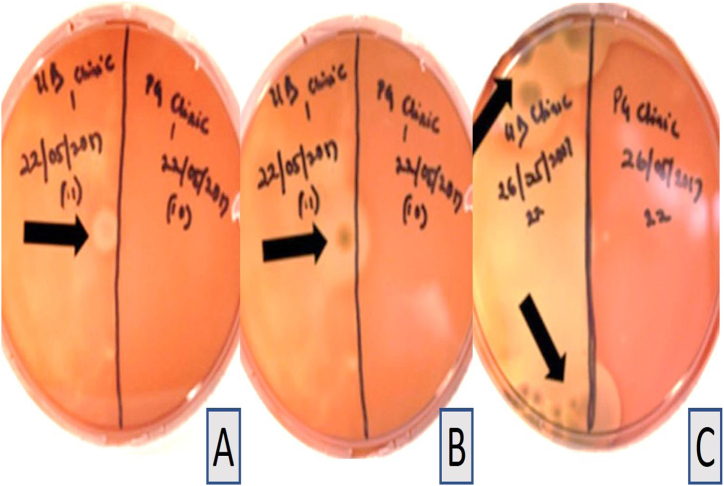
General dentistry teaching clinic sample inoculated on sheep blood agar at 37 °C. A, The first general dentistry teaching sample showing microbial growth found after 24 h. B, Further microbial growth of the same microbial colony after 3 days. C, The second general dentistry teaching clinic sample with microbial growth found after 48 h * The black arrows indicate the microbial growth.

All control sheep blood and tryptic soy agar plates prepared with the disinfectant without immersion of impressions and incubated at 37 °C, showed no growth after 24 h, one week, or 14 days (Table 1 and Fig. 1A–H). Haemolysis was observed in the pilot study at both 24 °C and 37 °C and was attributed to the concentration of the disinfectant. The four samples collected from the commercial dental laboratory after being used for immersion of impressions for one week showed no microbial growth on sheep blood agar or tryptic soy agar plates after 24 h or 14 days of incubation at 37 °C (Table 1 and Fig. 2A–H). Four samples were collected from the professional dental clinic after being used for immersion of impressions for one day only. The collected samples showed no microbial growth on sheep blood agar or tryptic soy agar plates after 24 h and 14 days of incubation at 37 °C (Table 1 and Fig. 3A–H). The prosthetic clinic samples that were collected after being used for immersion of impressions for one day only showed no microbial growth on sheep blood agar or tryptic soy agar plates after 24 h of incubation at 37 °C (Table 1 and Fig. 4A and B). After 72 h (3 days) of incubation, bacterial growth was found on two samples and continued to grow for 9 days (Fig. 4C–G). The remaining eight samples showed no microbial growth on sheep blood agar or tryptic soy agar plates after 14 days of incubation at 37 °C (Table 1). A culture revival streaked out on to a clean sheep blood agar and tryptic soy agar plates showed microbial growth after 24 h of incubation at 37 °C (Table 1 and Fig. 5A and B). Two out of six selective and differential media the bacteria were streaked on showed no microbial growth while the other four showed microbial growth (Fig. 5G and H). The colonies found showed an increase in size after two to four days (Table 1 and Fig. 5A–F). Thirteen samples were collected from the general dentistry teaching clinic after immersion of impressions for one day only. Eleven samples were processed and showed no microbial growth on sheep blood agar or tryptic soy agar plates after 24 h, one week or two weeks of incubation at 37 °C. The remaining two samples collected and incubated at 37 °C showed microbial growth on the sheep blood agar after 24–72 h of incubation (Table 1 and Fig. 6A–C). Culture revival made on sheep blood agar and tryptic soy agar plates showed microbial growth after 24 h of incubation (Table 1 and Fig. 6A–C). None of the plates of the tryptic soy agar incubated with the sheep blood agar during the primary isolation showed growth after 14 days except during culture revival. One of the isolated colonies showed an increase in size after 7 days (Table 1).

**Table 1:** summarizes the important findings of the study.

**Timing of appearance of bacterial colonies**	• Two to three days during primary isolation. • One day during culture revival.
**Size of bacterial colonies**	• Increased in size after two to four days. • One colony increased in size after seven days.
**Microscopic appearance**	• Gram + ve rod- shaped organisms with no visible spore. • Gram + ve rod-shaped organisms with sub terminal spore. • Gram -ve rod- shaped organisms with no spores in a cluster cell arrangement. • Gram variable (both positive and negative), rod- shaped organisms with spores with clusters multiple cells.
**Bacterial species**	• Common bacterial species that are part of the normal oral flora were not found. • Rare bacterial species as *Bacillus circulans, Bacillus horneckiae, Bacillus altidudinis or pumilus and Bacillus cereus* were isolated.
**Level of operator experience**	• No bacterial growth was found in samples collected from the professional dental clinic and the commercial dental laboratory. • Bacterial growth was found on some of the samples collected from teaching clinics.
**Frequency of replacement of the solution**	Following the manufacturer's instruction in replacement of the solution every week or when visibly contaminated provided satisfactory results.

### 4.2 Microscopic characteristics

**Fig. 7:**
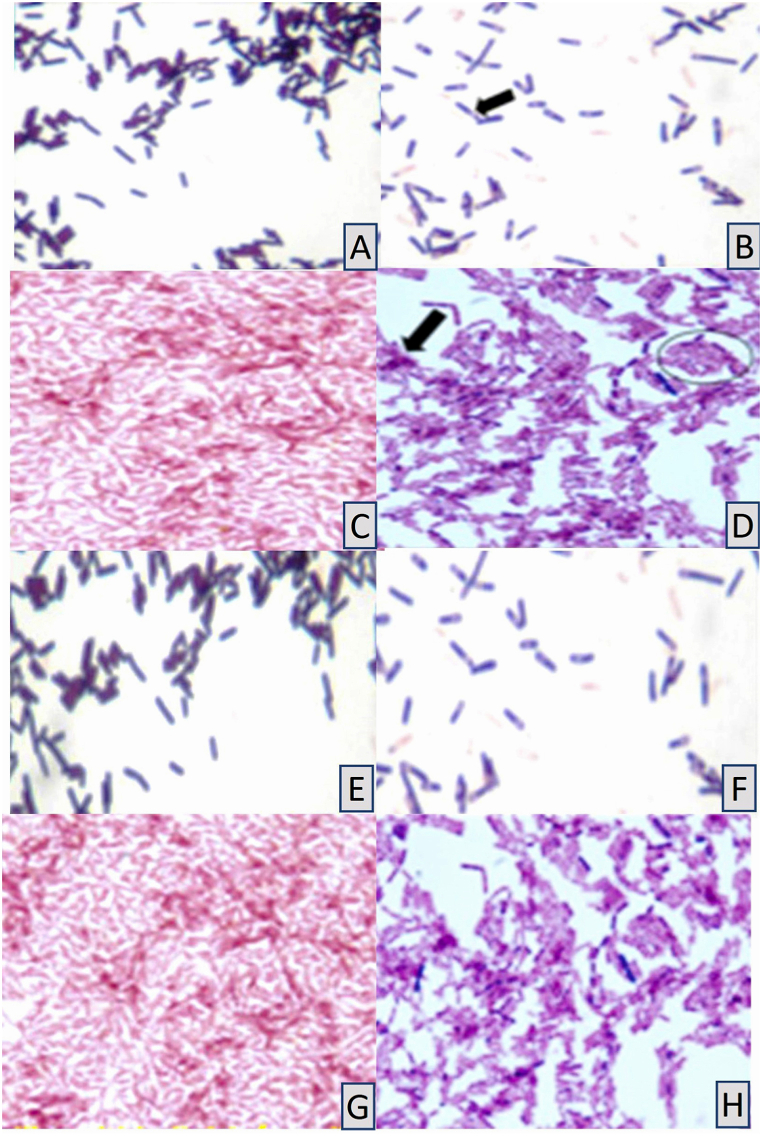
Microscopic features of the collected Cavex Impresafe samples. A, The first removable prosthetics teaching clinic sample. B, The second removable prosthetics teaching clinic sample, the purple color is an illustration of the gram + ve bacteria (gram stain X 100). C, The first general dentistry teaching clinic sample, the red colour is an illustration of the gram –ve bacteria (gram stain X100). D, The second general dentistry teaching clinic sample, the purple color is an illustration of the gram + ve bacteria (gram stain X100). * The black arrow indicates the variable stain (both positive and negative). * The black circle indicates how the cells are arranged in clusters. E-H, Show light microscopy picture of the Bacillus species MALDI-TOF MS identified in the Griffith University Dental Clinic samples. (X100).

The first sample from the prosthetics teaching clinic showed rod-shaped organisms with no visible spore when gram + ve staining was used (Table 1 and Fig. 7A). The second sample, when stained with Gram stain positive showed rod-shaped organisms with sub terminal spore (Table 1 and Fig. 7B). The first sample from the general dentistry teaching clinic, when stained with gram stain negative (-ve) showed rod-shaped organisms with no spores visible. The sample showed a cluster cell arrangement (Table 1 and Fig. 7C). The isolate from the second general dentistry teaching clinic sample was found to be gram variable stain (both positive and negative), rod-shaped with spores with clusters multiple cells (Table 1 and Fig. 7D).

### 4.3 MALDI-TOF MS identification

The automated machine identified the unknown microorganism isolated from the Cavex ImpreSafe disinfectant used in the Griffith University Dental Clinic as follows (Fig. 7A–H): First removable prosthetics teaching clinic sample = *Bacillus circulans*; second sample = *Bacillus horneckiae*. First General dentistry teaching clinic sample = *Bacillus altidudinis or pumilus* which could not be distinguished using the MALDI-TOF MS identification method; second sample = *Bacillus cereus* (Table 1).

## 5 Discussion

Data from the current study generally showed efficacy of Cavex ImpreSafe against common oral flora. These findings are supported by earlier studies [8,11,[17], [18], [19]]. The results, however, also show that some bacteria can survive in the Cavex ImpreSafe disinfectant with repeated use. This disproves that the disinfectant solution is sterile because several bacterial species are known to be resistant to benzalkonium chloride, including enterococci, *Klebsiella pneumoniae*, staphylococci and Pseudomonas spp [[20], [21], [22]]. The Bacillus species that were isolated from the used Cavex ImpreSafe disinfectant (i.e. *Bacillus circulans, Bacillus horneckiae, Bacillus altidudinis/pumilus and Bacillus cereus*) are not a common part of the oral flora. According to study by Logan, Old and Dick [23] *Bacillus circulans* is mostly found in soil, sewage, and food. It has been associated with wound infection [23], severe sepsis in an immunocompromised patient [24], prosthetic heart valve endocarditis [25] and cerebrospinal fluid shunt infection [26]. In addition, Alebouyeh et al. [24] suggested that *Bacillus circulans* should be treated as a potent pathogen for immunocompromised patients. A study by Shivaji et al. [27] noted the gene similarity of *Bacillus altitudinis* to *Bacillus pumilus*. *Bacillus altidudinis* was first isolated from cryogenic tubes used for collection of air from high altitudes [27], although its pathogenicity is unknown. A study by From, Hormazabal and Granum [28] noted *Bacillus pumilus* was found to be associated with food poisoning after it was found in rice. In addition, Tena et al. [29] found *Bacillus pumilus* to be associated with the development of cutaneous lesions similar to anthrax. Of the four Bacillus species that survive in Cavex ImpreSafe disinfectant, only *Bacillus cereus* has been associated with dental infection causing periodontal diseases [30]. *Bacillus cereus* is an opportunistic human and animal pathogen causing food poisoning and other severe infections [30].

An interesting finding from the current study is the isolation of *Bacillus horneckiae*, a novel Gram-positive aerobic bacterium that was first isolated from the Kennedy Space Centre. In 2008, it was named after the radiation biologist Dr. Gerda Horneck who discovered this previously unknown species of Bacillus and pioneered work exposing spores to space [31]. However, the pathogenicity of this bacteria is still unknown.

Comparing the professional dental clinic with the student teaching clinics, the student clinics showed higher levels of microbial contamination of the disinfectant solution. This could be contributed to the pre-rinsing practices of the impressions before decontamination. This step in the process described in the instructions might not have been uniformly followed in the students' clinics when compared to the professional dental clinic where a qualified dental assistant performs the rinsing step before the disinfection of the impressions. Furthermore, another contributing factor would be the larger number of impressions produced and immersed in the disinfectant solution per day in the students’ clinics. This could increase the microbial challenge to the disinfectant. Moreover, the commercial laboratory receives pre-processed impressions, which usually have been disinfected already. Following good practice, the laboratory disinfects all dental devices on receipt, not assuming they were fully decontaminated. Thus, it is likely the disinfectant solution is challenged with a much lower burden of microbes from impressions already disinfected.

From the results, it is clear to suggest that this Cavex ImpreSafe solution is likely effective against many commonly associated bacteria such as *Staphylococcus aureus, Pseudomonas aeruginosa and Enterococcus hirae* and not as effective against gram-positive rod-shaped bacteria (e.g., Bacillus species). This could be because of formation of impermeable spores [32,33] and the presence of the dipicolinic acid in the bacterial spores’ wall. This was supported by an earlier study by Slieman and Nicholson [34]. A study by Egusa et al. [8] found that 0.25 % of benzalkonium chloride was effective in removing oral pathogens but it was ineffective against biofilms of methicillin - resistant *Staphylococcus* [35]. It is reasonable to explain that benzalkonium chloride in high concentration (0.25 %) is effective enough for use as a disinfectant [6], as explained by the CDC 2008 which supported this explanation [36]. In addition, some researchers [8,11,18,37,38] found that benzalkonium chloride is bactericidal however has no activity against bacterial spores [37], which probably explains why it has such poor effectiveness against the Bacillus species. Many papers have demonstrated the efficacy of BAC against bacteria found in solutions sampled from impression materials [8,11,18,19]. Results from previous studies showed that impressions that were disinfected in the solution of BAC were later found to be effectively disinfected. In contrast, this study found significant amounts of growths of bacteria in solutions sampled from containers immersed with impression materials. This is in agreement with studies by Kaplan et al. [18], Egusa et al. [8], Oplinger and Wagner [19], Samra and Bhide [10] and Demajo et al. [11] who showed that bacteria could be isolated from disinfected samples. However, the previous studies were simultaneously testing the disinfectant solute ion, the method of disinfection, and the bacteria-harbouring properties of the impression material, respectively. Egusa et al. [8] evaluated a system of combined mechanical and chemical disinfection; other studies such as Kaplan et al. [18] compared spraying and dousing. The studies by Samra and Bhide [10] and Demajo et al. [11] commented on the tendency for alginate impressions to harbour several-fold more bacteria than silicone impression material. These variables compound the assessment of efficacy in studies where they were not controlled for or explicitly compared. A further variable that was not always described in the methods was how often the disinfectant solution was changed; whether it was used for multiple batches or only once. This study directly tested the integrity of the solution when used on multiple occasions, using a standard concentration. The results from this study were not reliant on the properties of the impression material or the method of utilisation. Thus, it was a more specific test of the disinfectant solution. The study by Oplinger and Wagner [19] is in one way more comparable to this study. It evaluated the repeated use of one batch of disinfectant, in this case over several months, for the disinfection of fishery equipment. The current study compared repeated use of BAC, in a real-world dental environment. Oplinger and Wagner [19] again used an indirect method for evaluating efficacy. Bacteria impregnated cards, using an isolated organism of interest, were disinfected then cultured, analogous to culturing a swab taken from the dental impression. That study showed efficacy against the desired organism, maintained by the solution for the life of the experiment.

A limitation of the current study was that the minimum inhibitory concentration (MIC) for benzalkonium chloride was not explored. The commercial product as tested relied on the manufacturer ‘s recommendations for reconstitution. It did not compare different concentrations and thus did not evaluate the inhibitory concentration. This study showed that using the manufacturer's recommendations, bacteria could survive. Specific tests to detect fungi and viruses were not performed, therefore this paper will not make any conclusions about the efficacy against these types of microorganisms based on the absence of growth of these organisms. There were no swabs taken for the patients intra-orally nor from the impressions before disinfection to determine the initial microbial load. This study also does not address the clinical implications of the survival rates of Bacillus species in dental settings. Neither does it address whether the organisms identified are pathogenic, nor if the isolated bacteria were growing or just surviving. Further information on clinical standards of disinfectants can be found in Guidelines [9,36,[39], [40], [41]].

Comparing the disinfectant from the current study with the effectiveness of Chlorhexidine in literature reveals different concentrations of Chlorhexidine ranging between 0.2 %–5 % has been proven effective to reduce biofilm with limited effect on Candida albicans as well as no dimensional changes on different impression materials [[42], [43], [44], [45]]. However, the recommended immersion time for Chlorhexidine is longer than Benzalkonium Chloride (10 min versus 3 min) [42]. Furthermore, Chlorhexidine had a long-term effect on the surface properties and hardness of silicone and acrylic resin [43,[46], [47], [48]]. However, a recent review and meta-analysis concluded that Chlorhexidine is recommended for longer and/or repeated immersions of resin denture bases [49]. On the other hand, alkaline peroxidase affected the surface roughness of resin denture bases, while sodium hypochlorite produces less changes on the surface of the denture bases [50]. A comprehensive study that investigated the effect of traditional disinfection methods (immersion and sprays) as well as modern disinfection methods (ultraviolet radiation and ozone) on the properties of different types and viscosities of silicone impression materials found that all methods had no significant effect on impression materials’ properties with the exception of some changes to the hardness [51]. Another factor that needs to be taken into consideration is the storage time between disinfection and pouring the impressions. It was reported that pouring impressions that were stored for one day after disinfection showed changes in dimensional accuracy that were within acceptable clinical ranges [52]. Further studies are required to provide sufficient data about the effects of benzalkonium chloride and other disinfecting methods on the properties of different dental materials [53]. A recent systematic review and meta-analysis concluded that a single disinfecting solution cannot be labelled as a universal disinfecting agent for all types of dental impression materials [54].

New advances like incorporation of silver nanoparticles into heat cured acrylic resin [55] and irreversible hydrocolloids [56] provides antimicrobial activity against common oral microbes like *S.mutants* without affecting the roughness of the material, however, 3D printed resin material showed less superior properties after impregnation with silver nanoparticles [55]. Furthermore, zinc oxide nanoparticles (ZnO-NP) or copper oxide nanoparticles (CuO-NP) were tested for their antibacterial activity within alginate impression materials and showed promising results without affecting the alginate's properties [57]. This opens the way for future breakthroughs in disinfection of dental materials using gaseous ozone [53,[58], [59], [60]], ultraviolet radiation [53,61] microwave therapy [60] and ultrasound treatment [62] as sustainable and environmentally friendly alternatives despite the fact that a published comprehensive review and comparison of disinfection techniques and protocols available in the literature concluded a few years ago that Iodophor is a recommended disinfecting solution for all types of impression materials [63].

The results obtained from the current study regards the effectiveness of Benzalkonium Chloride are backed by medical and dental literature as Benzalkonium Chloride was reported to be effective in disinfection of nasotracheal intubation [64], having an antibacterial effect in dental restorative materials [65] and being an effective ingredient in hand sanitizers against the SARS-CoV-2 virus, which was pivotal during the COVID-19 pandemic [66]. Our results demonstrated that a second level disinfection protocol in the commercial laboratory was essential to back up any deterioration of the efficacy of the disinfectant solution or imperfections in the disinfection protocol in the dental clinic. That addresses previous concerns raised in the literature regards cross-contamination of dental impressions and/or appliances in the dental laboratory environment [[67], [68], [69]]. A study that investigated the microbes isolated from the surfaces of dental laboratory equipment and dental laboratory staff attire showed the presence of strains that were not part of the normal oral flora as well as being resistant to antimicrobials [70]. Furthermore, studies have alerted about the levels of knowledge and compliance of impression disinfection and infection control practices amongst dental technicians [69,71]. The above findings illustrate that the type of the chemical disinfecting solution and the protocol are equally important in achieving optimum results [72]. It also highlights that further investigations are deemed necessary to inform clinicians about the best possible ways to ensure cross-contamination does not occur from the dental laboratory to the clinical environment when appliances are returned to be issued chairside.

The strengths of the current study are related to confirming the efficacy of the Cavex Impresafe disinfection solution when used according to manufacturer's guidelines. The present study also highlighted the importance of a second-level disinfection protocol in dental laboratories to compensate for any potential flaws in the disinfection protocols within dental clinics. The study also demonstrated the high variability of bacterial species that could be carried on and transmitted via oral appliances including *Bacillus horneckiae* isolated from a space centre and other species that are linked to food poisoning, cryogenic tubes used for collection of air from high altitudes and opportunistic infections in immunocompromised patients as well as around prosthetic valves and cerebrospinal fluid shunts; all of which are not part of the normal oral flora. The current study has some limitations including that fungal growth and viral species were not investigated in the disinfectant solution samples collected. Furthermore, it was not possible to standardize the number of impressions immersed in each sample, however, this simulates clinical scenarios in everyday practice and is in agreement with manufacturer's instructions that recommends replacement of the solution when it looks visibly contaminated. The current study did not compare different disinfectant solution concentrations and did not test the minimum inhibitory concentration of BAC. Finally, the present study was carried out in a university clinic and might not be representative of the entire population.

## 6 Conclusions

Cavex Impresafe is effective against common oral bacteria, when used according to the manufacturer's instructions in clinical practice. However, the solution cannot be considered sterile; rare bacterial species survived and could be cultured.

## Data availability statement

Data will be made available on request.

## CRediT authorship contribution statement

**Simon Chukwu:** Writing – original draft, Project administration, Methodology, Formal analysis, Data curation, Conceptualization. **Alan Munn:** Writing – review & editing, Validation, Supervision, Resources, Investigation. **Jennifer C. Wilson:** Writing – review & editing, Validation, Supervision, Investigation. **Hadeel Ibrahim:** Writing – review & editing, Writing – original draft, Validation, Investigation, Formal analysis. **Dean Gosling:** Writing – review & editing, Visualization, Resources, Methodology, Investigation, Data curation. **Robert M. Love:** Writing – review & editing, Validation, Supervision, Project administration, Funding acquisition, Conceptualization. **Mahmoud M. Bakr:** Writing – review & editing, Writing – original draft, Validation, Supervision, Project administration, Methodology, Funding acquisition, Formal analysis, Conceptualization.

## Declaration of competing interest

The authors declare that they have no known competing financial interests or personal relationships that could have appeared to influence the work reported in this paper.
